# Helicobacter Pylori Virulence Factor Cytotoxin-Associated Gene A (CagA) Induces Vascular Calcification in Coronary Artery Smooth Muscle Cells

**DOI:** 10.3390/ijms24065392

**Published:** 2023-03-11

**Authors:** Martin O. Sundqvist, Jonatan Wärme, Robin Hofmann, Sven-Christian Pawelzik, Magnus Bäck

**Affiliations:** 1 Department of Clinical Science and Education, Division of Cardiology, Karolinska Institutet, Södersjukhuset, SE-118 83 Stockholm, Sweden; martin.sundqvist.2@ki.se (M.O.S.); jonatan.warme@ki.se (J.W.); robin.hofmann@regionstockholm.se (R.H.); 2 Department of Cardiology, Södersjukhuset, SE-118 83 Stockholm, Sweden; 3 Translational Cardiology, Department of Medicine Solna, Karolinska Institutet, SE-171 77 Stockholm, Sweden; sven-christian.pawelzik@ki.se; 4 Department of Cardiology, Heart and Vascular Center, Karolinska University Hospital, SE-171 76 Stockholm, Sweden

**Keywords:** vascular calcification, *Helicobacter pylori*, cytotoxin-associated gene A, coronary artery smooth muscle cells, cardiovascular disease

## Abstract

*Helicobacter pylori* (*H. pylori*) has been associated with cardiovascular diseases. The pro-inflammatory *H. pylori* virulence factor cytotoxin-associated gene A (CagA) has been detected in serum exosomes of *H. pylori*-infected subjects and may exert systemic effects throughout the cardiovascular system. The role of *H. pylori* and CagA in vascular calcification was hitherto unknown. The aim of this study was to determine the vascular effects of CagA through human coronary artery smooth muscle cell (CASMC) osteogenic and pro-inflammatory effector gene expression as well as interleukin 1β secretion and cellular calcification. CagA upregulated bone morphogenic protein 2 (BMP-2) associated with an osteogenic CASMC phenotype switch and induced increased cellular calcification. Furthermore, a pro-inflammatory response was observed. These results support that *H. pylori* may contribute to vascular calcification through CagA rendering CASMCs osteogenic and inducing calcification.

## 1. Introduction

Vascular calcification, i.e., the deposition of hydroxyapatite within the vessel wall, plays an important role in the development of cardiovascular disease. The presence and extent of it is known to increase the risk of vascular events and overall mortality [1]. Coronary artery calcification is a central feature in the development of atherosclerosis and is a marker of cardiovascular outcomes, even in asymptomatic patients [2]. Alongside pathological states of hyperphosphatemia, as in chronic kidney disease [3], inflammation is a known factor for the initiation and development of vascular calcification [4]. Vascular smooth muscle cells (VSMC) play an integral part in the pathogenesis through various active processes [5]. Differentiation of VSMC into specific pathological phenotypes that favor processes of vascular calcification has been recognized, but underlying mechanisms are largely unknown [6].

The role of chronic infections in vascular calcification has not yet been firmly established. *Helicobacter pylori* (*H. pylori*) is a Gram-negative gastric bacterium that is known to cause gastritis, peptic ulcers, and gastric cancer [7]. *H. pylori* has been associated with several extra-gastric diseases, including cardiovascular disease [8,9,10], but its connection to vascular calcification is elusive. Active *H. pylori* infection in patients with acute myocardial infarction is more common in patients with the subtype of ST-elevation myocardial infarction compared with non-ST-elevation myocardial infarction [11], referring to the diagnostic appearance of an elevated ST-segment on electrocardiography that usually indicates complete coronary artery occlusion rather than a partial one [12]. One study compared coronary artery calcification scores assessed with computed tomography between *H. pylori*-seropositive and -seronegative healthy individuals and found an association with mild coronary artery calcification in the *H. pylori-*positive group, regardless of traditional risk factors [13]. Another smaller study with similar methodology found positive *H. pylori* serology to be more common in the higher levels of coronary artery calcification after multivariate adjustment [14]. A connection between the two could have large implications as vascular calcification is central in the development of cardiovascular disease, and at the same time *H. pylori* remains one of the most common chronic infections with an estimated global prevalence of around 50% [15]. However, the mechanism behind a potential causative relationship is unknown, and experimental studies are lacking. There are several suggested mechanisms by which *H. pylori* contributes to cardiovascular disease [9,10,16,17]. Some mechanisms have direct effects, such as an induction of a chronic, low-grade systemic inflammation, pro-thrombotic effects, and molecular mimicry between *H. pylori* antigens and proteins of the vasculature [18], whereas in others, the mechanism seems to be indirectly through an association with established risk factors such as dyslipidemia and dysregulation of glucose metabolism.

Interestingly, the presence of the *H. pylori* virulence factor cytotoxin-associated gene A (CagA) is also associated with cardiovascular disease [19,20,21]. CagA is a 120–145 kDa oncoprotein present in approximately 50–70% of all *H. pylori* strains [7]. It is a major virulence factor involved in the development of *H. pylori*-related gastric adenocarcinoma. CagA mediates a range of intra-cellular effects in host gastric epithelial cells by affecting different signaling pathways to promote inflammation, proliferation, motility, and changes in cellular polarity and, thus, increases the risk of neoplasia and gastritis [22]. Recently, one study revealed that CagA could be detected in exosomes in systemic circulation of *H. pylori*-infected patients. Furthermore, following induced CagA expression, gastric epithelial cells secrete CagA-containing exosomes [23], indicating that this pro-inflammatory virulence factor could reach distal organs through systemic circulation. The latter points to a possible route for *H. pylori*-caused extra-gastric diseases [24,25]. Considering the possibility of circulating CagA, the observations of *H. pylori* in vascular calcification and the known importance of VSMC in this process, the aim of this study was to determine the vascular effects of CagA. To this end, the effects of CagA on coronary artery smooth muscle cells (CASMCs) were determined and indicated that CagA mediates a pro-inflammatory and osteogenic response. 

## 2. Results

### 2.1. CagA Elicits an Inflammatory Response in CASMCs

**Figure 1:**
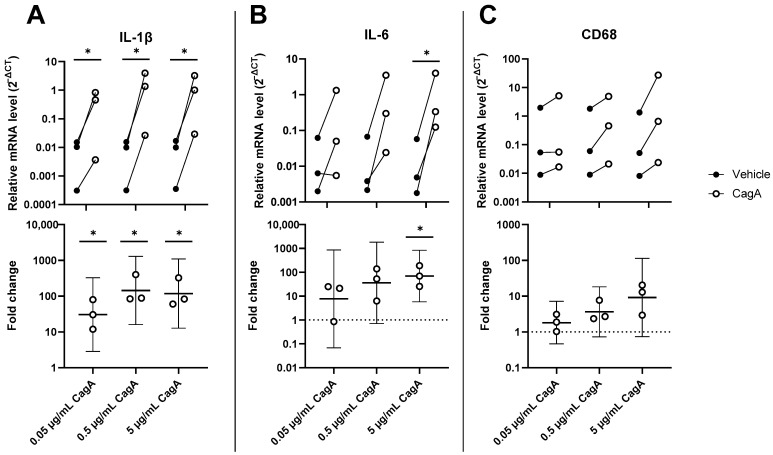
*H. pylori* virulence factor cytotoxin-associated gene A (CagA) upregulates expression of pro-inflammatory genes in coronary artery smooth muscle cells (CASMCs). mRNA levels of IL-1β (**A**), IL-6 (**B**), and CD68 (**C**) were analyzed by qPCR in CASMCs following stimulation with CagA. *n* = 3 with paired vehicle controls. Upper panels display relative expression to glyceraldehyde 3-phosphate dehydrogenase (GAPDH) calculated as 2^−ΔCT^. Lower panels show fold change with mean and 95% confidence intervals. * *p* < 0.05 vs. vehicle control (paired *t*-test).

To evaluate the effect of CagA on inflammation in CASMCs, mRNA levels of interleukin-1 beta (IL-1β), interleukin-6 (IL-6), and Cluster of Differentiation 68 (CD68) were determined by qPCR. IL-1β was significantly upregulated by CagA (Figure 1; 0.05 µg/mL, mean fold change 30.6, 95% CI 2.9 to 328, *p* = 0.03; 0.5 µg/mL, mean fold change 143.6, 95% CI 16.0 to 1287, *p* = 0.01; 5 µg/mL, mean fold change 117.6, 95% CI 12.7 to 1090, *p* = 0.01). IL-6 showed significant upregulation by CagA at 5 µg/mL (mean fold change 69.6, 95% CI 5.8 to 833, *p* = 0.02), but not at 0.5 µg/mL (mean fold change 35.9, 95% CI −1.4 to 1833, *p* = 0.06). CD68 was dose-dependently upregulated but did not reach significance (0.5 µg/mL, mean fold change 3.7 95% CI −1.4 to 18.3, *p* = 0.07); 5 µg/mL, mean fold change 9.2, CI −1.4 to 113, *p* = 0.06)).

**Figure 2:**
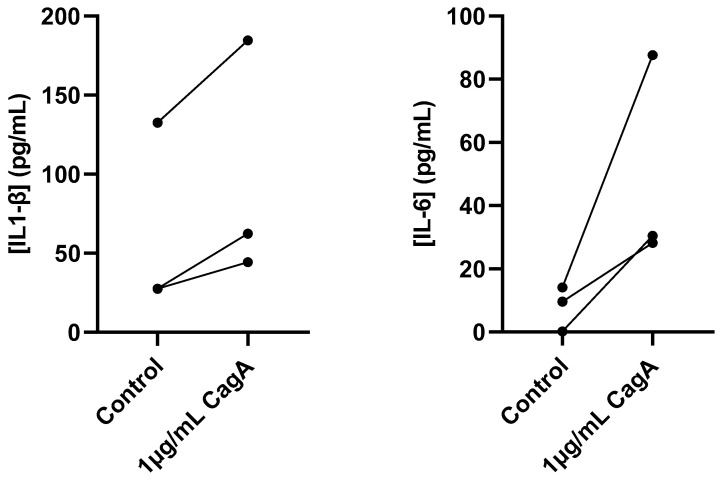
Cytotoxin-associated gene A (CagA) induces pro-inflammatory cytokine secretion in coronary artery smooth muscle cells (CASMCs). Concentrations of IL-1β and IL-6 in cell culture medium from CASMCs following 24 h induction with 1 µg/mL CagA were measured using ELISA. *n* = 3. *p* = 0.08 for IL-1β and *p* = 0.13 for IL-6 (paired *t*-test).

To further characterize the inflammatory response to CagA in CASMCs, secreted pro-inflammatory cytokines were quantified using ELISA in cell culture medium following treatment with 1 µg/mL CagA (Figure 2). Both IL-1β and IL-6 showed higher concentrations after CagA incubation, but the difference did not reach statistical significance (IL-1β, mean concentration of 68.3 pg/mL for control vs. 91.6 pg/mL for CagA treatment, *p* = 0.08; IL-6, mean concentration of 8.0 pg/mL for control vs. 48.8 pg/mL for CagA treatment, *p* = 0.13). Although cells from one donor showed higher baseline concentrations of IL-1β in the control group (133 pg/mL), than cells from the other donors CagA treatment further increased the IL-1β level to 185 pg/mL.

### 2.2. CagA Induces Cyclooxygenase 2 (COX-2) in CASMCs

**Figure 3:**
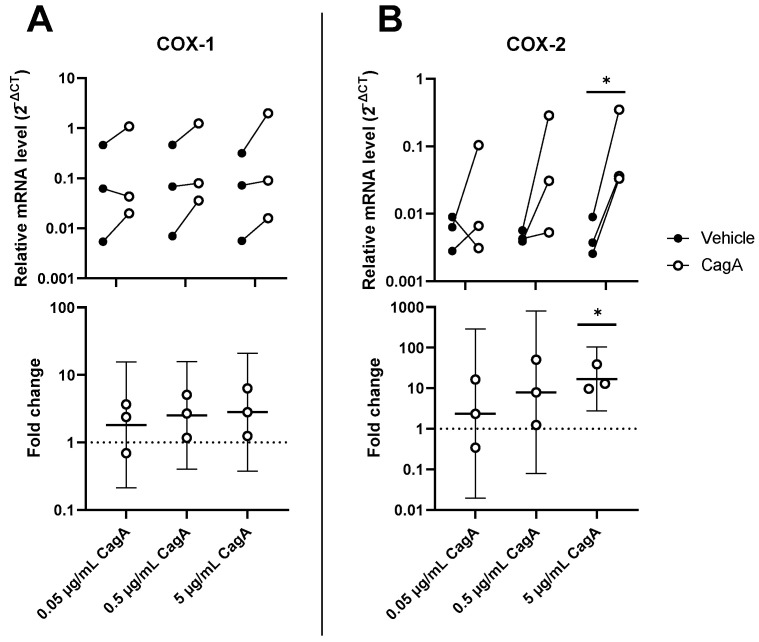
Cytotoxin-associated gene A (CagA) induces the cyclooxygenase (COX) pathway in coronary artery smooth muscle cells (CASMCs). Expression levels of prostanoid-synthesizing enzymes COX-1 (**A**) and COX-2 (**B**) were analyzed by qPCR following stimulation of CASMCs with CagA. *n* = 3 samples with paired vehicle controls. Upper panels display relative expression to glyceraldehyde 3-phosphate dehydrogenase (GAPDH) calculated as 2^−ΔCT^. Lower panels show fold change with mean and 95% confidence intervals. * *p* < 0.05 vs. vehicle control (paired *t*-test).

To further characterize the pro-inflammatory effects of CagA on CASMCs, mRNA levels of enzymes involved in the synthesis of prostanoids, i.e., cyclooxygenase 1 (COX-1) and COX-2 along with downstream prostanoid synthases, microsomal prostaglandin E_2_ synthase-1 (MPGES1), prostaglandin I_2_ synthase (PGIS), thromboxane A_2_ synthase (TXAS1), and prostaglandin D_2_ synthase (PTGDS) were determined. While COX-2 was significantly upregulated by CagA (mean fold change 16.9, 95% CI 2.7 to 104, *p* = 0.02), COX-1 showed increased expression, but results did not reach statistical significance (Figure 3). The expression of all other tested downstream prostanoid synthases were not significantly changed (Supplementary Materials, Figure S1).

### 2.3. CagA Induces Calcification in Vascular Smooth Muscle Cells

**Figure 4:**
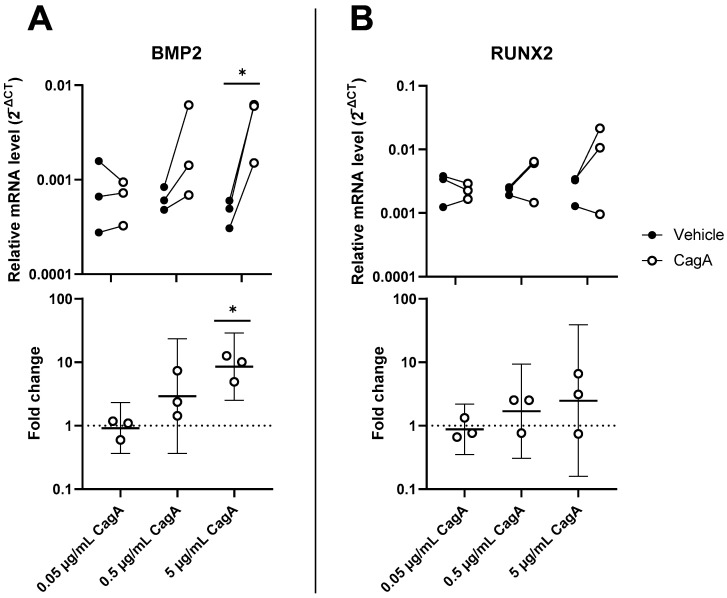
Cytotoxin-associated gene A (CagA) induces an osteogenic expression signature in coronary artery smooth muscle cells (CASMCs). Expression of major regulator genes of osteogenesis and calcification BMP2 (**A**) and RUNX2 (**B**) was measured by qPCR in CASMCs following CagA stimulation. *n* = 3 samples with paired vehicle controls. Upper panels display relative expression to glyceraldehyde 3-phosphate dehydrogenase (GAPDH) calculated as 2^−ΔCT^. Lower panels show fold change with mean and 95% confidence intervals. * *p* < 0.05 vs. vehicle control (paired *t*-test).

To explore the effect of CagA on vascular calcification, mRNA levels of Runt-related transcription factor 2 (RUNX2) and bone morphogenetic protein 2 (BMP2) were analyzed in CASMCs. Both mRNAs were increased in a dose-dependent way to the CagA challenge (Figure 4). BMP2 was significantly upregulated by 5 µg/mL CagA treatment (mean fold change 8.6, 95% CI 2.5 to 28.9, *p* = 0.02). RUNX2 was upregulated at the higher CagA concentrations tested but did not reach statistical significance.

**Figure 5:**
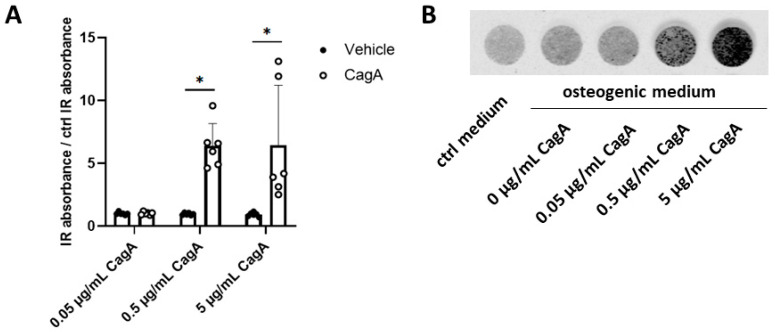
Cytotoxin-associated gene A (CagA) induces coronary artery smooth muscle cells (CASMCs) calcification. (**A**) Deposition of hydroxyapatite by CASMCs in the presence of osteogenic medium was quantified using the optical probe IRDye 800CW BoneTag. CagA increased calcification of CASMCs in a dose-dependent way. (**B**) Representative image of calcified CASMCs from one donor after five days of incubation with osteogenic medium and increasing concentrations of CagA. *n* = 3 biological replicates were used with 4 averaged technical replicates per sample. Two independent repeated experiments were performed, yielding 6 samples per treatment. Results were normalized for values obtained from CASMCs treated with control osteogenic medium and are displayed as mean with standard deviation. Statistical analysis was performed using two-way repeated-measures ANOVA followed by a post hoc Bonferroni test * *p* < 0.05 vs. vehicle control.

Finally, we evaluated CASMC calcification upon CagA challenge. CASMCs incubated with the two higher CagA concentrations significantly induced calcification (Figure 5).

## 3. Discussion

The results of this study point to effects of the *H. pylori* virulence factor CagA on CASMC inflammation and calcification. First, CagA increased pro-inflammatory cytokine transcription and secretion as well as a COX-2 induction. Furthermore, upregulation of the established key regulator of calcification, BMP2, by CagA was observed [6]. Finally, CagA increased calcification of CASMCs. These findings suggest pro-inflammatory and pro-calcific effects of CagA on CASMCs as a possible link between *H. pylori* infection and cardiovascular disease.

Observational studies have found an association between *H. pylori* and cardiovascular disease, commonly through sero-epidemiological methods. Patients with angina pectoris have higher titers of anti-CagA antibodies compared to healthy controls [21]. Meta-analyses show an increased risk of atherosclerosis [26] as well as cardiovascular diseases, e.g., ischemic stroke and myocardial infarction, among *H. pylori*-positive subjects [20,27]. *H. pylori*-specific DNA has been detected in coronary atherosclerotic plaques [28]. Interestingly, subgroup analysis shows a stronger association for patients infected with more virulent CagA-positive *H. pylori* strains [20]. Data on coronary artery calcification in relation to *H. pylori* are contradictory with positive associations reported [13,14], while some studies were neutral [29]. *H. pylori* could, thus, be a risk factor for the development of vascular calcification, particularly in the coronary arteries. It was hitherto not clear whether there is a causal relationship, and, if so, the underlying mechanisms involved. This study evaluated the *H. pylori* virulence factor CagA as one possible mechanistic factor for vascular calcification in *H. pylori*-infected patients. CagA induced an osteogenic CASMC phenotype switch, as supported by the upregulation of BMP2 and CagA-induced in vitro CASMC calcification. Furthermore, the upregulation of the macrophage marker CD68 by CagA, albeit not significant in the present study, may indicate induced CASMC expression of scavenger receptors and obtaining foam-cell-like characteristics [30]. A fraction of CD68-positive foam cells in atherosclerotic plaques in vivo also express specific markers of smooth muscle cells [31], indicating that CASMCs may play a direct role in inflammatory pathways by trans-differentiating into a macrophage-like phenotype, although the role of CagA in this process remains to be established.

The detection of CagA in serum exosomes of *H. pylori*-infected subjects represents a mechanism of how a gastric bacterial virulence factor could reach the distal coronary arteries to exert local effects in the heart [23,32]. A recent study also measured CagA in serum using an immunoassay, without first isolating exosomes, and reported circulating CagA in *H. pylori*-infected subjects [33], although the difference was not statistically significant from the control group. It remains unclear whether exosomes containing CagA derive from gastric epithelial cells after being infected via the type IV secretion system of *H. pylori* [22], whether they are directly derived from *H. pylori* bacteria, or both. It remains also to be determined how these exosomes enter systemic circulation. However, one study detected CagA in the vasa vasorum of human aortic sections from CagA-positive patients using immunohistochemical staining [34], which further indicates that systemic occurrence of CagA is possible and relevant in patients. In vivo mouse models using CagA-containing outer membrane vesicles of *H. pylori* as treatment accelerated atherosclerosis when administrated intra-gastrically [35].

CagA has been shown to accelerate atherosclerosis in mouse models, both through infection with CagA-positive *H. pylori* strains and through injection of recombinant CagA [36]. Other recent studies similarly show that CagA promotes not only atherosclerosis, but also endothelial dysfunction in mice. Infection with a CagA-positive *H. pylori* strain specifically determined the pathological effects in these experiments [37,38]. Our results show that CagA may not only be involved in cardiovascular disease by promoting atherosclerosis and affecting vascular endothelial cells but may also promote vascular calcification through effects on CASMCs. The finding in the present study that pro-inflammatory genes such as COX-2, IL-1β, and IL-6 are upregulated is in line with, and extends, similar findings presented previously using mouse aortic endothelial cells after treatment with recombinant CagA [39].

This is the first study showing direct effects of CagA on CASMCs. Certain limitations should, however, be acknowledged. The exact signaling mechanism by which CagA induces pro-calcific and pro-inflammatory pathways was not explored. Given the known, complex effects of CagA in gastric epithelial cells, several possible explanations can be considered. In these primary target cells of *H. pylori*, CagA affects several intracellular proteins and signaling pathways through phosphorylation-dependent and -independent processes. The phosphorylation-dependent effect requires CagA to interact with host tyrosine phosphatases, where CagA is phosphorylated at specific segments to enable further reactivity [40]. One interesting known interaction is the activation of tyrosine phosphatase SHP2 by CagA to deregulate Ras-ERK and promote malignancy. SHP2 is known to regulate BMP2 activation in osteoblasts [41], and similar effects could possibly be expected in CASMCs. Furthermore, CagA is pro-inflammatory and activates NF-kB both through the PI3K/Akt pathway as well as the STAT3/JAK2 pathway [40]. Both pathways have been shown to be activated in vascular endothelial cells following CagA stimulation [35,42]. The same pathways may have induced the pro-inflammatory effects in CASMCs observed in this study to stimulate their trans-differentiation toward an osteogenic and/or macrophage-like phenotype. Another limitation of this study is the limited sample size using CASMCs from three donors. We observed a large inter-donor variation of some studied responses, and even though several results reached statistical significance, statistical power may have been too low to detect smaller changes. The use of a fragmented recombinant protein is also a limitation, although above-mentioned mechanisms, and many other effects of CagA, lie near the end of the protein close to the c-terminal segment and are included in the used fragment [40]. 

The novel findings in the present study have the potential to stimulate future research. In particular, the role of *H. pylori* in vascular calcification remains incompletely understood in the context of mounting evidence regarding a connection to atherosclerosis. The limitations in the present study open the opportunity for extended studies to further characterize the observed responses. Moreover, future studies are needed to evaluate the in vivo effects of *H. pylori* CagA in vascular calcification, and clinical studies are required to evaluate an impact on cardiovascular outcomes.

In conclusion, this is the first study to show that CagA induces pro-inflammatory and pro-calcific effects on CASMCs through which *H. pylori* may induce vascular calcification and contribute to cardiovascular disease. Further studies are needed to explore how these findings translate to mechanisms underlying vascular calcification in vivo. The results also raise an initial notion for a therapeutic potential of *H. pylori* eradication for cardiovascular disease prevention.

## 4. Materials and Methods

### 4.1. Materials

Human coronary artery smooth muscle cells (CASMCs) from 3 donors were commercially obtained (CC-2583; Lonza, Basel, Switzerland). Cell culture reagents, i.e., Dulbecco’s modified Eagle’s medium (DMEM), fetal bovine serum (FBS), phosphate-buffered saline (PBS), Penicillin/Streptomycin, Amphotericin B, l-glutamine, HEPES, and trypsin were obtained from ThermoFisher Scientific, Waltham, MA, USA. Recombinant CagA protein containing amino acids 918 to 1147 and an N-terminal His tag was purchased from Abcam, Cambridge, UK. Reagents for the isolation of total RNA were purchased from Qiagen, Hilden, Germany. qPCR reagents were purchased from ThermoFisher Scientific, Waltham, MA, USA. ELISA kits for human IL-1β (DY201) and IL-6 (DY206-05) were purchased from R&D Systems, Minneapolis, MN, USA. IRDye 800CW BoneTag Optical Probe was purchased from LI-COR Biosciences, Lincoln, NE, USA.

### 4.2. Cell Culture

CASMCs were cultured in DMEM supplemented with 10% FBS, 100 units/mL penicillin, 100 μg/mL streptomycin, 2.5 µg/mL Amphotericin B, 1 mM sodium pyruvate, 10 mM HEPES, and 2 mM L-glutamine. Medium was changed every other day, and cells were regularly examined under a light microscope to assess for growth, morphology, and confluency. All cells were used at a passage number < 8. Prior to experimental treatment, cells were detached from their culturing flasks using trypsin, and approximate cell concentrations were measured using a Luna 2 cell counter (Logos Biosystems, Anyang, Republic of Korea). In this study, 120,000 cells per well were seeded into 12-well plates for qPCR and ELISA experiments, and 6000 cells per well were seeded into 96-well plates for calcification assays. Cultures were incubated in a humidified incubator at 37 °C with 5% CO_2_. Cells from three donors were used as biological replicates throughout all experiments. Two of the donors were of male and one donor was of female sex. All donors were age-matched and in a range between 12 and 22 years.

### 4.3. Treatment with H. pylori Virulence Factor CagA

Serial dilutions of recombinant CagA were prepared in DMEM supplemented with 5% FBS, 100 units/mL penicillin, 100 μg/mL streptomycin, 2.5 µg/mL Amphotericin B, 1 mM sodium pyruvate, 10 mM HEPES, and 2 mM l-glutamine for all experiments in this study. Concentrations of 5 µg/mL, 0.5 µg/mL, and 0.05 µg/mL were used as previously described by Yang et al. [36]. Cytokine secretion was measured at a single treatment concentration of 1 µg/mL CagA. Cells were treated for 24 hours for qPCR and ELISA or for 6 days for the calcification assays. Paired vehicle controls consisting of 50% glycerol and 50% tris-buffer, diluted in the same 5% FBS medium as CagA, were used for each treatment concentration in the qPCR and calcification assays.

### 4.4. qPCR

Following induction with CagA, cells were lysed and RNA was purified using RNeasy mini kit according to the manufacturer’s protocol. RNA concentrations were measured using a NanoPhotometer NP80 (Implen, Westlake Village, CA, USA). Total RNA was reverse-transcribed into cDNA using the high-capacity RNA-to-cDNA kit (ThermoFisher Scientific, Waltham, MA, USA), which was used as template DNA for quantitative polymerase chain reaction (qPCR) analysis on a QuantStudio 7 Flex system (ThermoFisher Scientific, Waltham, MA, USA). TaqMan Gene Expression Assays with the following Assay ID were used for transcriptional analysis: RUNX2, Hs01047973_m1; BMP2, Hs00154192_m1; PTGS1, Hs00377726_m1; PTGS2, Hs00153133_m1; PTGES, Hs00610420_m1; PTGIS, Hs00919949_m1; TBXAS1, Hs01022706_m1; HPGD, Hs00183950_m1; IL1β, Hs01555410_m1; IL6, Hs00985641_m1; CD68, Hs00154355_m1. Each sample was measured in three technical replicates. The relative mRNA expression of the target genes was quantified using the ΔC_T_ and ΔΔC_T_ method with Glyceraldehyde 3-phosphate dehydrogenase (GAPDH) as housekeeping gene (assay ID Hs99999905_m1).

### 4.5. ELISA

Conditioned medium from CASMCs induced for 24 h with 1 µg/mL CagA was used to study secretion of IL-1β and IL-6 and analyzed in technical duplicates by commercial ELISA kits according to the manufacturer’s protocol. Absorbance was measured at 450 nm wavelength and 540 nm as background reference on a Victor Spectrophotometer (PerkinElmer, Waltham, MA, USA) using SoftMax Pro, version 7.1, and concentrations were calculated using 4-parametric logistic regression.

### 4.6. Calcification Assay

Cells from *n* = 3 biological replicates were measured in quadruplicate technical replicates repeated two times to assess calcification as previously described [43]. An amount of 6000 cells per well were seeded in black 96-well plates and cultured for 6 days in osteogenic medium consisting of DMEM supplemented with 5% FBS, 100 units/mL penicillin, 100 μg/mL streptomycin, 2.5 µg/mL Amphotericin B, 1 mM sodium pyruvate, 10 mM HEPES, 2 mM l-glutamine, and 2.6 mM inorganic phosphate (P_i_) to facilitate calcification, or in regular medium as non-osteogenic control. CagA or vehicle (50% glycerol and 50% tris-buffer) was added to the osteogenic medium at the indicated concentrations. After 5 days of culture, 10 nM IRDye 800CW BoneTag Optical Probe was added to the medium and incubated for another 24 hours. Following incubation, wells were rinsed twice with PBS and then filled with 150 µL of PBS. Calcification was then assessed qualitatively and quantitively using the imaging platform Odyssey CLx (LI-COR Biosciences, Lincoln, NE, USA). Calcification was quantified as arbitrary units of intensity and normalized to the intensity of the osteogenic control for each donor.

### 4.7. Statistics

For qPCR, one experiment was performed for each sample and treatment concentration. Samples were pipetted in triplicates and results were averaged. ΔC_T_ values were calculated for treatment and matched vehicle control for each treatment concentration, and paired *t*-tests were used to determine significance with a *p*-value < 0.05 considered significant. A paired *t*-test comparing treatment and control was used for ELISA experiments. For calcification assays, absorbance values were normalized to cells treated with control osteogenic medium. Data were then analyzed using a two-way repeated-measures ANOVA on averaged values of technical replicates and repeats for each treatment per sample. A post hoc test was performed comparing treatment means, with Bonferroni multiple testing correction. All statistical analysis was conducted using Prism version 8 (GraphPad, San Diego, CA, USA) and Sigmaplot version 15 (Systat Software Inc., San Jose, CA, USA).

## Data Availability

Individual data that underlie the results reported will be shared with researchers who provide a methodologically sound proposal. Investigators interested in data should contact the corresponding author.

## Supplementary Materials

The following supporting information can be downloaded at: https://www.mdpi.com/article/10.3390/ijms24065392/s1.
