# Supplementary material for: Helicobacter Pylori Virulence Factor Cytotoxin-Associated Gene A (CagA) Induces Vascular Calcification in Coronary Artery Smooth Muscle Cells
Source: Int J Mol Sci. 2023 Mar 11;24(6):5392. doi: 10.3390/ijms24065392 (PMC10049385; doi:10.3390/ijms24065392)
Supplement: Supplementary file 1 [file ijms-24-05392-s001.zip › ijms-2256308-supplementary.pdf]

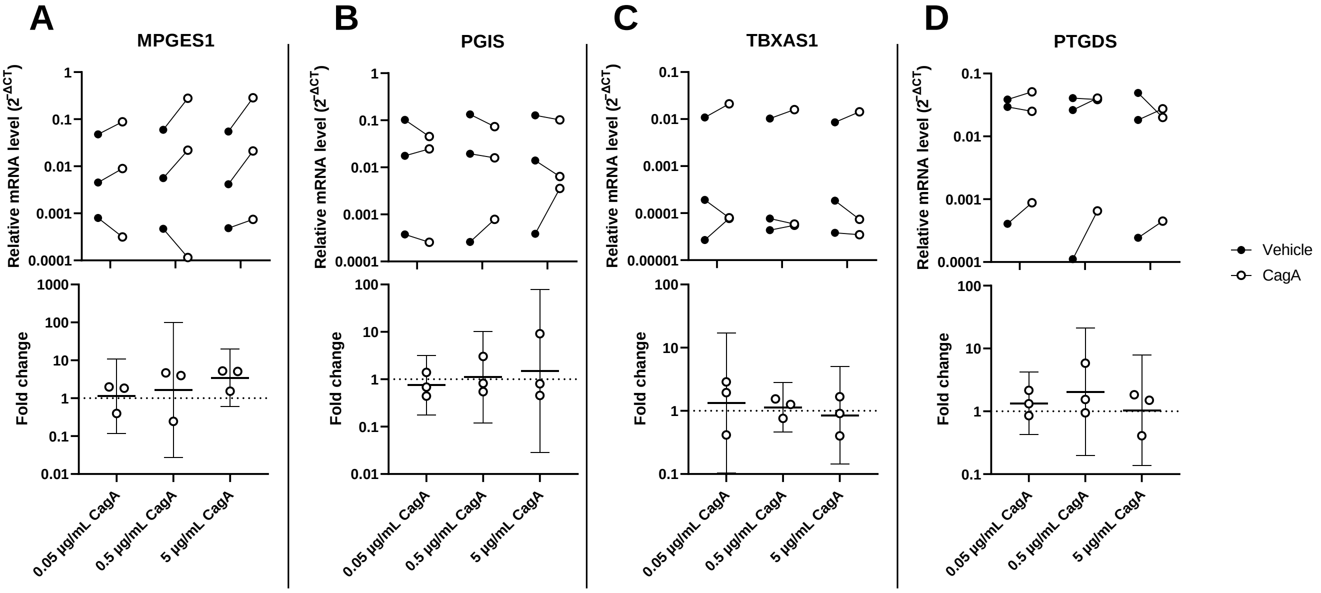

Supplemental Figure S1. Expression of downstream prostanoid synthases in coronary artery smooth muscle cells (CASMCs) stimulated by Cytotoxin-associated gene A (CagA)

Expression of microsomal prostaglandin E_2 synthase-1 (MPGES1) **(A),** prostaglandin I_2 synthase (PGIS) **(B)**, thromboxane A_2 synthase (TXAS1) **(C)**, and prostaglandin D_2 synthase (PTGDS) **(D)** was measured by qPCR in CASMCs following CagA stimulation. *n* = 3 samples with paired vehicle controls. Upper panels display relative expression to glyceraldehyde 3-phosphate dehydrogenase (GAPDH) calculated as $2^{-\Delta CT}$. Lower panels show fold change with mean and 95% confidence intervals.
